# Social support and quality of life in migrant workers: Focusing on the mediating effect of healthy lifestyle

**DOI:** 10.3389/fpubh.2023.1061579

**Published:** 2023-03-23

**Authors:** Yufan Yang, Shuzhen Zhao, Lulu Lin, Jieyu Qian, Haiyan Zhang, Fuman Cai

**Affiliations:** ^1^The Second School of Medicine, Wenzhou Medical University, Wenzhou, China; ^2^The Second Affiliated Hospital and Yuying Children's Hospital of Wenzhou Medical University, Wenzhou, China; ^3^Division of Epidemiology and Health Statistics, Department of Preventive Medicine, School of Public Health and Management, Wenzhou Medical University, Wenzhou, China; ^4^College of Nursing, Wenzhou Medical University, Wenzhou, China

**Keywords:** social support, quality of life, migrant worker, lifestyle, mediating effect

## Abstract

**Objective:**

To investigate the relationship between social support and quality of life of Chinese migrant workers and to explore the mediating role of healthy lifestyles in social support and quality of life.

**Methods:**

Using a stratified multi-stage sampling method, 1, 298 migrant workers and 983 urban workers across 110 neighborhood committees in five economic development zones in eastern China were surveyed. The social support level of participants was quantified using the Social Support Rating Scale, and quality of life was evaluated using the SF-8. Healthy lifestyle was evaluated based on a combination of sleep, smoking, alcohol consumption, and exercise. Multiple linear regression analysis was used to assess the relationship between quality of life and social support. Stepwise regression was used to analyze the mediating effect of healthy lifestyle, social support, and quality of life among migrant workers.

**Results:**

Total SSRS and total SF-8 scores of migrant workers were significantly higher than those of urban workers (*P* < 0.001). After controlling for confounders, social support showed an independent positive association with quality of life for both migrant (β = 0.50, *P* < 0.05) and urban workers (β = 0.62, *P* < 0.05). Mediation effect analysis revealed that healthy lifestyle partially mediated the relation between social support and quality of life of migrant workers with a mediation effect of 0.07, accounting for 11.70% of the total effect.

**Conclusions:**

This study showed a significant correlation between social support and quality of life of Chinese migrant workers, with healthy lifestyle playing a mediating role. Improving the social support and health literacy of migrant workers and developing a healthy lifestyle are key to improving their quality of life.

## 1. Introduction

Migrant workers, also known as rural-to-urban migrant workers, constitute a unique social group in China, referring to those who have rural *hukou* and work in non-agricultural jobs in cities and towns. *Hukou*, also known as household registration, is a proof of identity for every Chinese citizen that is used to record and retain basic information about the resident population. Every Chinese citizen is classified as either rural *hukou* or urban *hukou* depending on the area of residence and is granted certain benefits and rights depending on the *hukou*. In other words, urban benefits and rights are only available to urban residents. Therefore, migrant workers are deprived of certain rights of urban citizens because they do not have urban *hukou* irrespective of the duration of their stay in the city, which will also lead to their lower status than urban residents (1–3). Owing to the rapid economic development and urban-rural integration in China, the number of migrant workers is rapidly increasing. According to the National Bureau of Statistics of China, the number of migrant workers in 2021 was 5.4% higher than that in 2015, reaching a total of 293 million, more than 1/5 of China's total population (4). A large number of migrant workers migrated from rural areas to cities to work in the construction industry. It is undeniable that these migrant workers cater to the large workforce requirement for urban development, and also promote the development of tertiary industries such as service catering, and play an important role in the rapid development of China's economy and urbanization (1, 5–7). Migration from rural to urban areas can improve the living and financial conditions of migrant workers, moreover, they can enjoy the convenience of urban public service facilities, which inculcates a certain sense of happiness and satisfaction (1, 8). However, due to the lack of urban *hukou*, migrant workers do not have access to the same rights and benefits and social welfare resources as urban residents (3, 9). In addition, the hardship and lack of job security, discrimination experiences, difficult cultural adaptation process, social inequality, and weak social support system can cause mental health problems such as depression among migrant workers, which can adversely affect their health and quality of life (10–14).

The World Health Organization defines quality of life as the impact of patient-reported medical conditions on functional health and wellbeing, including physical, mental/emotional, and social aspects (15). Social support has been shown to be an important moderator of stress relief and coping with poor health outcomes, while poorer social support is associated with reduced quality of life (16, 17). Helgeson's research revealed a deep relationship between social support and quality of life. When stress levels are low, individuals, as part of a social network, can feel surrounded by friends and family, etc., which can help them maintain a better quality of life. Conversely, when stress levels are high, individuals need to improve their quality of life by using resources in their social network to help cope with stress and frustration (18). Studies have also shown that owing to the migration status, migrant workers tend to experience health-related problems that lead to poor quality of life, moreover, both the original social support networks of migrant workers change significantly due to the fundamental changes in the form of employment and life space patterns brought about by migration (19, 20). Studies have shown that social support is an important factor that influences the health of migrant workers, in addition, good social support often plays a positive role in promoting or protecting their physical and mental health, and can also significantly alleviate migration-related stress (21, 22). On the other hand, a healthy lifestyle can significantly reduce the incidence and mortality of certain diseases and play an important role in maintaining health and improving the quality of life of individuals (23, 24). A study of the relationship between social support, loneliness, physical activity, and quality of life in Korean older adults found that physical activity (as an important component of lifestyle) had a significant positive effect on quality of life (25). In addition, a study in China found that all dimensions of social support were significant predictors of health-promoting lifestyles, meaning that increasing the level of social support significantly improved individuals' lifestyles (26). Therefore, based on these findings, we speculated that social support may influence individual's lifestyle and thus quality of life. However, no studies have reported on the association between social support, healthy lifestyle, and quality of life of Chinese migrant workers. Therefore, this study aimed to investigate the current status of social support, healthy lifestyle, and quality of life among Chinese migrant workers and explore their relationships. Based on the literature, we hypothesized that social support and quality of life are significantly related, and a healthy lifestyle plays a mediating role.

## 2. Materials and methods

### 2.1. Ethical consideration

This study was approved by the Ethics Committee of the Wenzhou Medical University (Protocol No. 2019079). All participants were aware of the purpose of the study and signed an informed consent form. The right to voluntary participation in the study, confidentiality, and anonymity were guaranteed.

### 2.2. Settings and participants

China is divided into eastern, central, western, and northeastern regions based on the differences in economic development and geographical location (4). Compared to other regions, the eastern region of China has a developed economy and a large base of migrant workers. Considering the accessibility of the study population, this cross-sectional study was conducted from August 2019 to January 2020 using a multi-stage stratified sampling method in a total of 110 neighborhood committees in five economic development zones in eastern China, including Suzhou, Jiangsu Province, Shanghai, Wenzhou, Zhejiang Province, Xiamen, Fujian Province, and Shenzhen, Guangdong Province. The purpose of this study was to investigate the quality of life and social support status of migrant workers and urban workers, respectively. Migrant workers were defined as workers with household registration in rural areas and who were engaged in non-agricultural industries locally or outside the home for ≥6 months. Urban workers were defined as those who had urban household registration and were practicing and living in the local area. To improve the comparability of the study population, migrant workers and urban workers were selected separately from the same workplace or neighborhood committee, rather than randomly from the general population.

All enumerators underwent a standardized training before the survey and were allowed to conduct the questionnaire survey only after passing the training. A pilot survey of 30 migrant workers and 30 urban workers was conducted separately before the formal survey to understand the acceptance level of the questionnaire by the typical respondents. This allowed for adjustments and improvements in the formal survey based on the feedback. All participants were asked to complete a standardized questionnaire to obtain general demographic information, while the quality of life of the subjects was investigated using the short form (SF-8) Health Survey and the level of social support was assessed using the Social Support Rating Scale (SSRS).

### 2.3. Measures

A self-administered standardized questionnaire was used to collect basic sociodemographic information of participants, including age, gender, education level (including education below high school, high school and above high school), marital status (including married and unmarried), occupation (including work stability, nature of work and job level), economic level (based on annual household income per capita), lifestyle, and so on.

#### 2.3.1. Social support

The Social Support Rating Scale (SSRS) (27) was used to evaluate the level of social support among migrant and urban workers. The scale contains a total of 10 items encompassing three dimensions of social support i.e., objective support, subjective support, and availability of social support. Objective support refers to the support from friends, family and social networks that can meet the physical, psychological and social needs of the individual. Subjective support refers to moral support such as respect, understanding, and acceptance from family members such as parents, siblings, classmates, and friends. Availability of social support refers to the extent to which individuals utilize and participate in social support when they experience frustration. The total score of SSRS ranged from 12 to 66, with higher scores indicating better levels of social support for the subjects. The score of SSRS between 12–22 is considered to have poor social support, 23–44 is considered to have moderate social support, and 45–66 is considered to have adequate social support (28). The SSRS has been widely used to measure the level of social support in the Chinese population (29–31). The Cronbach's α value of the SSRS in this study was 0.77.

#### 2.3.2. Quality of life

SF-8 was used to assess the quality of life of migrant workers and urban workers (15). Compared with the SF-36 and SF-12, SF-8 has the shortest entries and content. Moreover, SF-8 has the advantages of simplicity and requires shorter survey time, making it suitable for use in large-scale surveys (15). SF-8 contains a total of eight items, each corresponding to a dimension of quality of life, including general health perception (GH), physical functioning (PF), social functioning (SF), role limitations due to physical health problems (role-physical, RP), bodily pain (BP), energy/fatigue (vitality, VT), role limitations due to emotional problems (role-emotional, RE), and psychological distress and wellbeing (mental health, MH). The final summary is divided into two sections, the Physical Component Summary (PCS) and the Mental Component Summary (MCS). The original score for each entry is converted to a standardized score ranging from 0 to 100, Physical Component Summary (PCS) and the Mental Component Summary (MCS) as well as the total score are calculated according to the formula in the SF-8 manual. A higher score is considered to be a better quality of life for the subject (32). The Cronbach's α value for the SF-8 in this study was 0.88.

#### 2.3.3. Healthy lifestyle

Healthy lifestyle scores base on sleep, smoking, alcohol consumption, and exercise, were constructed according to the relevant recommendations of the World Health Organization (33). All lifestyle factors were obtained through a self-reported structured questionnaire. Healthy sleep was defined as early to bed and early to rise; no history of consumption of alcohol and smoking was defined as healthy levels; ≥3 times per week of heavy exercise was rated as healthy. Healthy level was assigned a score of 1 while 0 was assigned for other conditions. Scores for sleep, smoking, alcohol consumption, and exercise were added to calculate the healthy lifestyle score.

### 2.4. Data analysis

Statistical Analysis System (SAS) was used to create a database and perform statistical analysis. Data were independently entered by two researchers followed by crosschecking and verification to minimize errors. Questionnaires with ≥20% missing content were considered invalid and excluded. Descriptive analyses of general demographic information, quality of life, and level of social support were performed. Normally distributed continuous variables were presented as mean ± standard deviation while non-normally distributed continuous variables were presented as median and quartiles. Categorical variables were presented as frequency (percentage). The Chi-squared test or Fisher's exact test was used to assess the differences in the study variables between the migrant and urban worker groups. Multiple linear regression analysis was used to assess the relationship between quality of life and social support among migrant workers and urban workers, respectively. The mediating effects were analyzed for migrant workers' healthy lifestyles, social support, and quality of life based on the stepwise regression method of testing the causal effects of mediating variables proposed by Baron et al. (34).

## 3. Results

### 3.1. Socio-demographic characteristics

**Figure 1:**
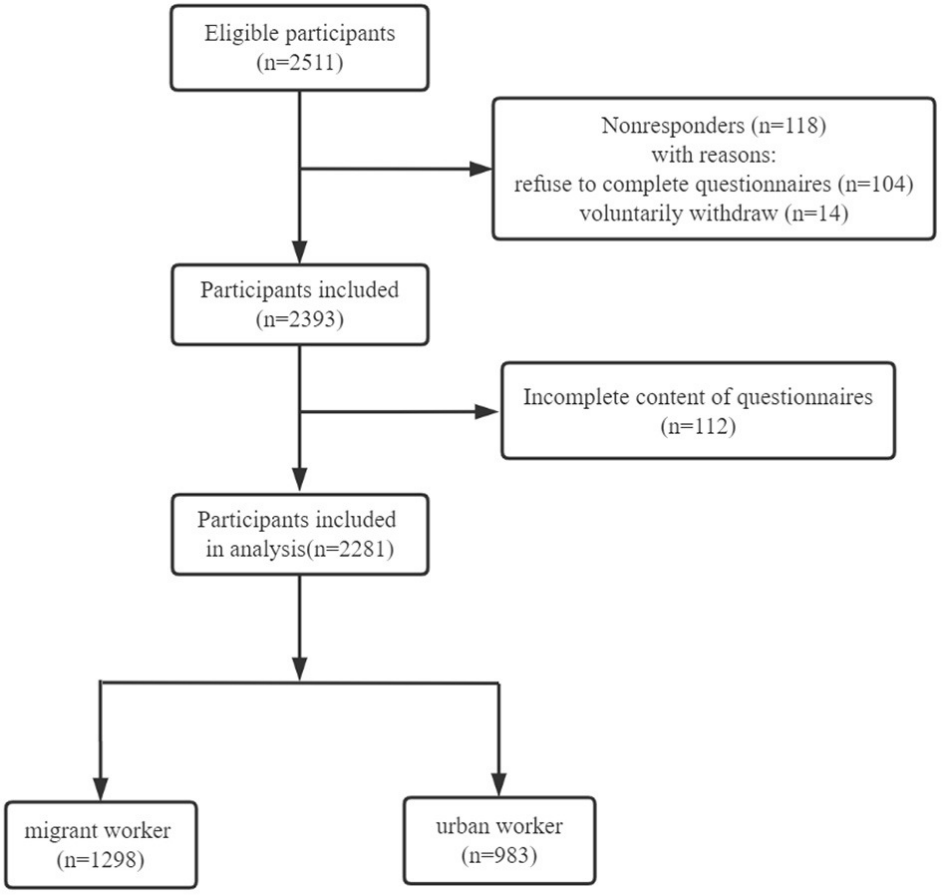
Flow chart of participant selection.

Of the 2, 511 questionnaires distributed, 118 participants voluntarily withdrew during filling of the questionnaires. In addition, 112 questionnaires were excluded due to incomplete content (≥20% missing information). Finally, a total of 2, 281 participants (983 urban workers and 1, 298 migrant workers) participated in the study. The flow chart of participants selection was shown in Figure 1. There was no significant difference between migrant and urban workers with respect to age. More than half of the participants, both migrant and urban workers, were male, and almost all were of Han Chinese ethnicity. In terms of place of residence, migrant workers were significantly more likely to rent or live in a dormitory (*P* < 0.001). The proportion of married migrant workers was significantly higher than that of married urban workers (*P* < 0.001). Migrant workers had significantly lower levels of education than urban workers (*P* < 0.001). The median income of urban workers was greater than that of migrant workers (*P* < 0.05). In addition, migrant workers are more likely to be in debt than urban workers and less likely to have deposits than urban workers (*P* < 0.001). Regarding the nature of work, migrant workers were more likely to engage in physical labor, while urban workers were more likely to engage in non-physical work (*P* < 0.05). The socio-demographic characteristics of migrant and urban workers are summarized in Table 1.

**Table 1:** Sociodemographic characteristics of the study population.

**Characteristics**	**Overall (*N* = 2, 281) *n* (%)**	**Urban workers (*N* = 983) *n* (%)**	**Migrant workers (*N*= 1, 298) *n* (%)**	***P*-value**
**Age [y, Mean (SD)]**	35.7 ± 11.4	35.3 ± 10.8	36.0 ± 11.8	0.136
**Gender**				0.992
Male	1, 309 (57.39)	564 (57.38)	745 (57.40)	
Female	972 (42.61)	419 (42.62)	553 (42.60)	
**Ethnicity**				0.713
Han Chinese	2, 183 (95.70)	939 (95.52)	1, 244 (95.84)	
Ethnic minorities	98 (4.30)	44 (4.48)	54 (4.16)	
**Residence**				<0.001
Renting or dormitory	1, 495 (65.54)	419 (42.62)	1, 076 (82.90)	
Self-purchased houses	786 (34.46)	564 (57.38)	222 (17.10)	
**Marital status**				<0.001
Unmarried	755 (33.10)	379 (38.56)	376 (28.97)	
Married	1, 472 (64.53)	584 (59.41)	888 (68.41)	
Widowed	16 (0.70)	8 (0.81)	8 (0.62)	
Divorced	32 (1.40)	11 (1.12)	21 (1.62)	
Separated	6 (0.26)	1 (0.10)	5 (0.39)	
**Education**				<0.001
<High school	977 (42.83)	388 (39.47)	589 (45.38)	
High school	762 (33.41)	309 (31.43)	453 (34.90)	
>High school	542 (23.76)	286 (29.09)	256 (19.72)	
**Income, 10, 000 CNY/year [M (P25, P75)]**	7.00 (5.00, 10.00)	7.00 (5.00, 10.00)	6.00 (5.00, 10.00)	0.003
**Debts and deposits**				<0.001
Neither one	841 (38.45)	319 (33.65)	522 (42.13)	
Debts	544 (24.87)	227 (23.95)	317 (25.59)	
Deposits	802 (36.67)	402 (42.41)	400 (32.28)	
**Religious belief**				<0.001
No	1, 363 (59.75)	465 (47.30)	898 (69.18)	
Yes	918 (40.25)	518 (52.70)	400 (30.82)	
**Work stability**				0.020
Temporary	309 (14.59)	116 (12.55)	193 (16.16)	
Stationary	1, 809 (85.41)	808 (87.45)	1, 001 (83.84)	
**Nature of work**				0.003
Physical labor	762 (33.41)	316 (32.15)	446 (34.36)	
Mental work	457 (20.04)	229 (23.30)	228 (17.57)	
Both	1, 062 (46.56)	438 (44.56)	624 (48.07)	
**Job level**				<0.001
Ordinary staff	1, 577 (69.14)	646 (65.72)	931 (71.73)	
Management staff	411 (18.02)	236 (24.01)	175 (13.48)	
Head of Enterprise	293 (12.85)	101 (10.27)	192 (14.79)	
**Alcohol consumption**				0.582
Never	1, 238 (54.27)	540 (54.93)	698 (53.78)	
Yes	1, 043 (45.73)	443 (45.07)	600 (46.22)	
**Sleep habits**				0.308
Early to bed and rise	1, 014 (44.45)	425 (43.23)	589 (45.38)	
Non-early to bed and rise	1, 267 (55.55)	558 (56.77)	709 (54.62)	
**Smoking**				0.027
Never	1, 407 (61.68)	581 (59.10)	826 (63.64)	
Yes	874 (38.32)	402 (40.90)	472 (36.36)	
**Sports**				0.455
<3 times/week	1, 772 (77.69)	771 (78.43)	1, 001 (77.12)	
≥ 3 times/week	509 (22.31)	212 (21.57)	297 (22.88)	

CNY, China Yuan, $US1 = CNY6.95.

Data were presented as mean and standard deviation [Mean (SD)] for normal or similar normal distributed variables, number and percentage [*n* (%)] for categorical variables, or median (25th, 75th percentage) [M (P25, P75)] for variables having skewed distribution.

### 3.2. Social support between migrant and urban workers

**Figure 2:**
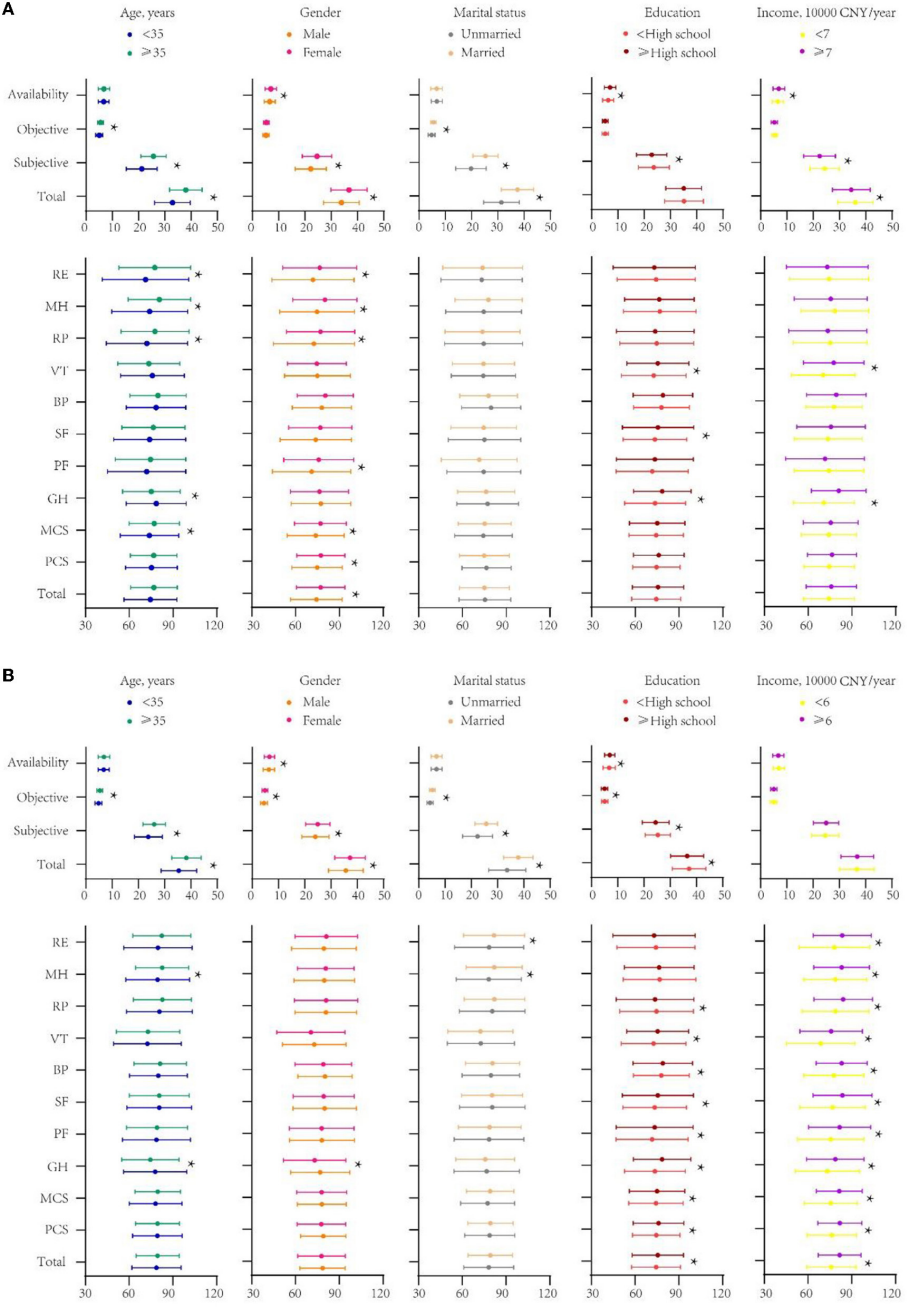
**(A)** Scores of social support and quality of life of urban workers by different sociodemographic variables. **(B)** Scores of social support and quality of life of migrant workers by different sociodemographic variables. *indicates *p* < 0.05. CNY, China Yuan, $US1 = CNY6.95; PF, Physical functioning; RP, Role physical; BP, Bodily pain; GH, General health; SF, Social functioning; VT, Vitality; MH, Mental health; RE, Role emotional; PCS, physical component summary; MCS, mental component summary.

The total SSRS score of migrant workers was significantly higher than that of urban workers (*P* < 0.001). In terms of different dimensions of social support, subjective support scores of migrant workers were higher than those of urban workers (*P* < 0.001). However, objective support scores of migrant workers were lower than those of urban workers (*P* < 0.001). There was no significant difference between the two groups in terms of availability of social support (*P* = 0.109) (Table 2). Migrant workers aged ≥35 years had higher total social support scores than those aged <35 years (*P* < 0.05) while similar results were observed in terms of subjective and objective support. The social support level of female migrant workers, including all dimensions, was significantly better than that of male migrant workers (*P* < 0.05). Married migrant workers had significantly higher total social support scores, subjective support, and objective support scores than unmarried migrant workers (*P* < 0.05). In addition, migrant workers with less than high school education had higher total, subjective and objective support scores than those with high school education and above, however, they had lower availability of social support (*P* < 0.05). Finally, the level of social support among migrant workers with different incomes did not show significant differences either in total score or in subjective support, objective support and availability (*P* > 0.05). Analysis of social support scores based on different sociodemographic variables is presented in Figure 2.

**Table 2:** Comparison of the scores for social support and quality of life between migrant and urban workers.

**Variables**	**Total (*N* = 2281)**	**Urban workers (*N* = 983)**	**Migrant workers (*N* = 1, 298)**	***P*-value**
**Social support**				
Total score	36.1 ± 6.7	35.1 ± 7.0	36.8 ± 6.4	<0.001
Subjective	24.1 ± 5.4	23.2 ± 5.9	24.9 ± 5.0	<0.001
Objective	5.1 ± 1.2	5.2 ± 1.2	5.1 ± 1.2	<0.001
Availability	6.8 ± 2.1	6.8 ± 2.2	6.9 ± 2.1	0.109
**Quality of life**				
Total score	77.3 ± 16.6	75.2 ± 17.3	78.9 ± 15.9	<0.001
PCS	77.6 ± 16.6	75.5 ± 17.0	79.2 ± 16.0	<0.001
Physical functioning (PF)	76.1 ± 24.0	72.7 ± 25.8	78.7 ± 22.2	<0.001
Role physical (RP)	78.3 ± 23.8	74.1 ± 26.1	81.5 ± 21.3	<0.001
Bodily pain (BP)	79.6 ± 19.4	78.6 ± 19.9	80.4 ± 19.0	0.034
General health (GH)	76.2 ± 20.6	76.6 ± 20.3	76.0 ± 20.8	0.560
MCS	77.0 ± 17.9	74.8 ± 18.8	78.6 ± 16.9	<0.001
Social functioning (SF)	78.0 ± 22.4	74.7 ± 23.4	80.4 ± 21.4	<0.001
Vitality (VT)	73.3 ± 22.1	74.4 ± 21.6	72.5 ± 22.5	0.046
Mental health (MH)	79.0 ± 22.2	76.6 ± 24.3	80.8 ± 20.2	<0.001
Role emotional (RE)	77.7 ± 24.8	73.5 ± 27.6	80.8 ± 21.8	<0.001

PCS, physical component summary; MCS, mental component summary.

### 3.3. Quality of life between migrant and urban workers

The quality of life of migrant workers and urban workers was significantly different in terms of total score and scores for all dimensions (*P* < 0.05) except general health (GH) (Table 2). Specifically, the quality of life of migrant workers was better than that of urban workers in terms of total score, PCS (including PF, RP, BP), and MCS (including SF, MH, RE) (*P* < 0.05). However, urban workers scored significantly higher than migrant workers on only one dimension, i.e., VT (*P* < 0.05). We next analyzed the quality of life scores of migrant workers disaggregated by sociodemographic variables. No significant differences (*P* > 0.05) were observed in the total SF-8 scores of migrant workers disaggregated by age, gender, and marital status; however, differences were observed only for individual dimensions. In terms of the total score, PCS (including PF, RP, BP, GH), and MCS (including SF, VT), the quality of life of migrant workers with less than high school education was lower than that of migrant workers with high school education and above (*P* < 0.05). In addition, the quality of life of migrant workers with income ≥60, 000 CNY was significantly better than that of migrant workers with income <60, 000 CNY in both total scores and all dimensions (*P* < 0.05). Detailed data is presented in Figure 2.

### 3.4. Multiple linear regression analyses of social support and quality of life

The relationship between social support and quality of life was assessed using multiple linear regression analyses. After controlling for age, gender, ethnicity, residence, marital status, education, income, debts and deposits, religion, job stability, job nature, work level, sleep habits, alcohol consumption, smoking, and exercise, the total scores of social support and quality of life showed an independent positive correlation in both migrant (β = 0.50, *P* < 0.05) and urban workers (β = 0.62, *P* < 0.05). Specifically, total social support (including subjective support, objective support) showed a significant positive correlation with total quality of life (including PCS, and MCS) for migrant workers; however, the availability of social support showed no significant correlation with quality of life (including PCS, and MCS). For urban workers, all dimensions of social support showed a significant positive correlation with quality of life (including PCS, and MCS). More details of the multiple linear regression analysis for each dimension of social support and each dimension of quality of life for migrant and urban workers are shown in Table 3.

**Table 3:** Multiple linear regression analyses of social support and quality of life among migrant and urban workers.

**Social support**	**Total**	**PCS**	**MCS**	**GH**	**PF**	**SF**	**BP**	**VT**	**RP**	**MH**	**RE**
**Urban workers**											
Total score	**0.62 (0.44, 0.79)**	**0.58 (0.41, 0.75)**	**0.66 (0.47, 0.84)**	**0.40 (0.19, 0.62)**	**0.66 (0.40, 0.92)**	**0.68 (0.44, 0.92)**	**0.51 (0.30, 0.72)**	**0.49 (0.26, 0.73)**	**0.74 (0.47, 1.00)**	**0.69 (0.45, 0.94)**	**0.76 (0.48, 1.03)**
Subjective	**0.47 (0.25, 0.69)**	**0.43 (0.21, 0.65)**	**0.51 (0.27, 0.75)**	**0.37 (0.10, 0.64)**	**0.44 (0.10, 0.77)**	**0.50 (0.19, 0.80)**	**0.37 (0.10, 0.63)**	**0.51 (0.22, 0.81)**	**0.54 (0.20, 0.88)**	**0.47 (0.16, 0.78)**	**0.57 (0.22, 0.92)**
Objective	**2.16 (1.18, 3.14)**	**2.07 (1.11, 3.04)**	**2.25 (1.17, 3.32)**	−0.92 (−2.11, 0.26)	**3.50 (2.04, 4.95)**	**2.88 (1.52, 4.23)**	**2.41 (1.24, 3.58)**	−1.03 (-2.38, 0.31)	**3.30 (1.82, 4.79)**	**3.27 (1.89, 4.66)**	**3.88 (2.33, 5.43)**
Availability	**1.85 (1.39, 2.32)**	**1.79 (1.32, 2.25)**	**1.92 (1.41, 2.43)**	**1.52 (0.94, 2.09)**	**2.01 (1.29, 2.72)**	**2.01 (1.35, 2.66)**	**1.47 (0.91, 2.04)**	**1.49 (0.85, 2.14)**	**2.16 (1.44, 2.88)**	**2.17 (1.51, 2.84)**	**2.00 (1.25, 2.76)**
**Migrant workers**											
Total score	**0.50 (0.35, 0.64)**	**0.45 (0.30, 0.60)**	**0.55 (0.39, 0.70)**	**0.51 (0.32, 0.70)**	**0.41 (0.20, 0.62)**	**0.54 (0.34, 0.74)**	**0.50 (0.33, 0.68)**	**0.47 (0.26, 0.68)**	**0.38 (0.18, 0.58)**	**0.56 (0.38, 0.75)**	**0.62 (0.42, 0.82)**
Subjective	**0.67 (0.49, 0.85)**	**0.62 (0.43, 0.81)**	**0.72 (0.52, 0.91)**	**0.71 (0.47, 0.95)**	**0.56 (0.30, 0.83)**	**0.72 (0.46, 0.97)**	**0.67 (0.44, 0.89)**	**0.56 (0.29, 0.83)**	**0.54 (0.29, 0.79)**	**0.73 (0.49, 0.96)**	**0.87 (0.62, 1.13)**
Objective	**1.30 (0.52, 2.07)**	**1.09 (0.30, 1.87)**	**1.51 (0.68, 2.33)**	0.31 (−0.73, 1.34)	**1.79 (0.68, 2.90)**	**2.05 (0.98, 3.11)**	0.64 (−0.32, 1.59)	0.28 (−0.86, 1.42)	**1.61 (0.55, 2.66)**	**1.80 (0.81, 2.80)**	**1.90 (0.82, 2.98)**
Availability	0.34 (−0.08, 0.76)	0.25 (−0.17, 0.68)	0.43 (−0.02, 0.87)	0.51 (−0.04, 1.06)	0.00 (−0.60, 0.60)	0.24 (−0.34, 0.81)	**0.58 (0.08, 1.09)**	**0.93 (0.33, 1.53)**	−0.09 (−0.65, 0.48)	0.43 (−0.10, 0.96)	0.11 (−0.47, 0.70)

Model controlled for age, gender, ethnicity, residence, marital status, education, income, debts and deposits, religious belief, work stability, nature of work, job level, sleep habits, drinking, smoking, and sports.

PF, Physical functioning; RP, Role physical; BP, Bodily pain; GH, General health; SF, Social functioning; VT, Vitality; MH, Mental health; RE, Role emotional; PCS, physical component summary; MCS: mental component summary.

Numbers indicate beta coefficients and 95% confidence intervals in multiple regressions, bolded indicates *p* < 0.05.

### 3.5. Analysis of the mediating effect of healthy lifestyle between social support and quality of life among migrant workers

Multiple linear regressions of social support and quality of life were first constructed based on unadjusted healthy lifestyle scores; further adjustment of healthy lifestyle scores revealed a large beta change between the two (*P* < 0.001), suggesting that healthy lifestyle scores may play a mediating role in the relation between social support and quality of life. Mediation effect analysis showed that healthy lifestyle partially mediated the relation between social support and quality of life of migrant workers, with a mediation effect of 0.07 and a ratio of 11.70% of the total effect. The specific mediating effect test is shown in Table 4.

**Table 4:** Proportion of association of social support with quality of life mediated by healthy lifestyle among migrant workers.

**Social support**	**No interaction**				**Interaction**			
	**Total effect**	**NDE**	**NIE**	**Percentage mediated**	**Total effect**	**NDE**	**NIE**	**Percentage mediated**
**Total**								
Total score	0.56 (0.43, 0.70)	0.50 (0.36, 0.64)	0.07 (0.03, 0.10)	**11.63 (5.62, 17.64)**	0.56 (0.42, 0.70)	0.49 (0.35, 0.63)	0.07 (0.03, 0.10)	**11.70 (5.64, 17.77)**
Subjective	0.76 (0.59, 0.94)	0.68 (0.50, 0.86)	0.09 (0.04, 0.13)	**11.15 (5.46, 16.85)**	0.76 (0.58, 0.93)	0.67 (0.49, 0.85)	0.08 (0.04, 0.12)	**11.17 (5.43, 16.91)**
Objective	1.11 (0.34, 1.88)	1.15 (0.39, 1.90)	−0.04 (−0.18, 0.10)	−3.36 (−16.67, 9.96)	1.07 (0.30, 1.84)	1.11 (0.35, 1.87)	−0.03 (−0.16, 0.09)	−3.05 (−15.18, 9.08)
Availability	0.49 (0.08, 0.89)	0.36 (−0.05, 0.76)	0.13 (0.05, 0.21)	**26.73 (0.78, 52.67)**	0.49 (0.08, 0.90)	0.36 (−0.05, 0.76)	0.13 (0.05, 0.21)	**26.90 (0.87, 52.93)**
**PCS**								
Total score	0.51 (0.37, 0.65)	0.45 (0.30, 0.59)	0.06 (0.03, 0.09)	**12.02 (5.34, 18.70)**	0.51 (0.36, 0.65)	0.45 (0.30, 0.59)	0.06 (0.03, 0.09)	**12.04 (5.33, 18.74)**
Subjective	0.71 (0.53, 0.89)	0.63 (0.45, 0.81)	0.08 (0.04, 0.12)	**11.15 (5.03, 17.27)**	0.70 (0.52, 0.88)	0.62 (0.44, 0.80)	0.08 (0.04, 0.12)	**11.16 (5.00, 17.33)**
Objective	0.90 (0.12, 1.68)	0.94 (0.16, 1.71)	−0.03 (−0.16, 0.10)	−3.82 (−19.16, 11.52)	0.87 (0.08, 1.65)	0.89 (0.12, 1.67)	−0.03 (−0.14, 0.08)	−3.44 (−17.32, 10.44)
Availability	0.37 (−0.04, 0.79)	0.25 (−0.16, 0.66)	0.12 (0.04, 0.20)	32.43 (−6.70, 71.55)	0.38 (−0.04, 0.79)	0.25 (−0.16, 0.66)	0.12 (0.04, 0.20)	32.85 (−6.34, 72.04)
**MCS**								
Total score	0.62 (0.47, 0.77)	0.55 (0.40, 0.70)	0.07 (0.04, 0.10)	**11.32 (5.52, 17.12)**	0.61 (0.46, 0.76)	0.54 (0.39, 0.69)	0.07 (0.04, 0.10)	**11.43 (5.54, 17.32)**
Subjective	0.82 (0.63, 1.01)	0.73 (0.54, 0.92)	0.09 (0.05, 0.14)	**11.15 (5.49, 16.81)**	0.81 (0.62, 1.00)	0.72 (0.53, 0.91)	0.09 (0.05, 0.13)	**11.18 (5.46, 16.89)**
Objective	1.32 (0.50, 2.14)	1.36 (0.55, 2.16)	−0.04 (-0.19, 0.11)	−3.04 (-15.03, 8.95)	1.28 (0.46, 2.10)	1.32 (0.51, 2.13)	−0.04 (-0.17, 0.10)	−2.79 (-13.79, 8.22)
Availability	0.60 (0.16, 1.03)	0.46 (0.03, 0.89)	0.14 (0.05, 0.23)	**23.18 (2.65, 43.72)**	0.60 (0.16, 1.03)	0.46 (0.03, 0.89)	0.14 (0.05, 0.23)	**23.15 (2.62, 43.67)**

Model controlled for age, gender, ethnicity, residence, marital status, education, income, debits and deposits, religious belief, work stability, nature of work, job level.

PCS, physical component summary; MCS, mental component summary; NDE, natural direct effect; NIE, natural indirect effect. Total Effect = NDE + NIE. The bold values indicate the value of *p* < 0.05.

## 4. Discussion

Currently, there is a paucity of data on the social support and quality of life of migrant workers in China. This study investigated and compared the social support and quality of life of migrant workers and urban workers from the same community or workplace and found some interesting phenomena. Our findings suggested that migrant workers had significantly better levels of social support than urban workers and showed similar phenomena in terms of quality of life. The social support and quality of life of migrant workers showed a significant correlation, and a healthy lifestyle was found to play a mediating effect.

Social support is a system of moral or material help and support provided to individuals by members within a social network. Good social support can improve an individual's self-confidence, ability to resolve negative emotions, and mental health, which in turn improves the quality of life (35). Our results revealed an interesting phenomenon, contrary to expectations, migrant workers had significantly better levels of social support than urban workers, both in terms of total social support and subjective support; however, the results were reversed in terms of objective support and there was no significant difference in terms of availability. This is contrary to the results of another study (19). The difference in age of the study population may explain this difference. Our study focused on migrant workers of all ages, while Xing et al. focused only on the new generation of migrant workers (aged 15–30 years). Studies have shown that the resources available to the migrant workers, such as social networks and information sharing channels, gradually increase with age, leading to a significant increase in social support (36). The superior social support of migrant workers compared to urban workers may be related to the following factors. When migrant workers go from familiar rural areas to unfamiliar cities to work, their employment information and resources often originate from family members, relatives, friends, or hometowns; therefore, it is easier to establish social support networks based on relatives, friends, and hometowns after arriving in cities. Moreover, they show the characteristics of residential aggregation and frequent interaction, and the traditional close hometown affection can still be continued (37). Therefore, subjective support, which is mainly emotional support, is not significantly affected and may even be enhanced. On the contrary, urban workers may have less access to social support channels than migrant workers, and thus have lower levels of social support. We also found that among the migrant workers, social support was better for participants who were aged ≥35 years, female, married, and with less than high school education. A previous study showed that women tended to have larger social support networks and are more likely to have frequent contact with relatives, friends, and colleagues for social support compared to men (38). At the same time, the typical female attributes of warmth, compassion, expressiveness, and family relationships may be expressed in increased social support (39). Second, marital relationships are one of the most important social relationships for individuals, and studies have shown that married people have better social support than unmarried people (40). Family members are a source of continuous support for individuals, and intimate relationships among family members can increase the amount and type of support received (41). Harmonious marriage is an important spiritual support and motivation for migrant workers to live and work in an unfamiliar city, which helps to relieve and regulate life stress and negative emotions; thus, the intimacy and warmth brought by marriage helps migrant workers to obtain more social support. Finally, we also found that migrant workers with less than high school education had better social support. However, contrary results were obtained on the dimension of availability of social support. This interesting finding was inconsistent with the results of other studies (42, 43). Migrant workers with lower education levels lagged behind those with higher education levels in both their perception of urban life and their ability to acquire and learn new skills, thus showing lower availability of social support, while better total social support instead may be caused by other confounding factors, which needs to be verified by further research.

Our findings also suggested that migrant workers had a significantly better quality of life than urban workers and that the results were consistent for physical component summary (PCS) and mental component summary (MCS). Clearly, this is an interesting finding. Previous studies have found that migrant workers who leave the familiar countryside to work and live in more competitive cities may experience more work-related stress and inadequate social support, leading to poorer mental health and quality of life (19, 20). However, our study yielded very different results, and the quality of life of migrant workers may not be as poor or even better than expected, which may be mediated by the following factors. First, the “healthy immigrant effect” at least partly explain this phenomenon, which refers to the fact that immigrants have, on average, better physical and mental health compared to natives (44, 45). Specifically, individuals who are willing to migrate for work tend to be better prepared, healthier, and more psychologically resilient to meet the demands of high-intensity work and unfamiliar urban environment (44). Secondly, increased income of migrant workers can help improve their family's financial situation, thus improving their psychological health and quality of life. However, some urban workers may feel dissatisfied with their low income compared to other urban residents, which can negatively affect their psychological health and quality of life (1, 31, 46). Furthermore, we found that the quality of life of migrant workers was better in those with high school and above education and among those with monthly income ≥60, 000 CNY, and there was no significant effect of age, gender, or marital status in this respect, which was generally consistent with the results of a previous study (47). Differences in quality of life are likely to result from differences in health literacy. Studies have shown that health literacy and personal health status are closely related to the quality of life (48). Compared to highly educated individuals, those with lower education levels have lesser ability to use various information to maintain a healthy state. On the other hand, individuals with lower income may have a more stressful life, which limits their ability to spend enough time to maintain their health status, and are less likely to be in a position to improve their life status, resulting in a poor quality of life (49).

Multiple linear regression analysis showed a significant positive correlation between social support and quality of life of migrant worker. This means that individuals with better social support are more likely to have a better quality of life. This was consistent with the results of other studies (19, 50, 51). Studies have found that social support, as a moderator or resilience factor in the face of unavoidable stress, has the potential to improve health-related outcomes and quality of life (52–54). Migrant workers leave their familiar rural environment to work in cities, and despite the improved income, the hardship of work, inadequate social security, difficulties in cultural adaptation, and discrimination tend to deeply trouble migrant workers (11, 12), affecting their psychological health and quality of life. Adequate social support may help migrant workers to cope with challenges positively and thus improve their quality of life. Studies have shown that individuals with healthy lifestyle tend to have a better quality of life as. Maria et al. (55) investigated the quality of life and lifestyle of 1, 968 men and 1, 737 women aged 18–90 years; they found that poor lifestyle was significantly associated with unhealthy behaviors and lower quality of life. Studies conducted in the United Kingdom (56) and Sweden (57) also supported the results. A Danish study found a significant negative association of unhealthy lifestyle with physical health and mental health. A healthy lifestyle such as improved dietary habits and increased exercise can significantly improve both physical health and mental health (58). Our study also found that a healthy lifestyle played a mediating effect in the relationship between social support and quality of life of migrant workers. This may be because migrant workers have more social support from relatives, friends, and workers in their social network, which greatly enhances their ability to face challenges; thus, they are more likely to be psychologically resilient, and desist from adopting unhealthy lifestyles (such as smoking) in the face of adversity, thus contributing to the improvement of their quality of life (1).

Migrant workers play a crucial role in China's economic development. Due to the special nature of this group, it is important to improve their social support and quality of life. First, it is particularly important to improve objective support for migrant workers. In recent years, although China has incorporated work injury insurance, pension insurance, and medical insurance in the social security system for migrant workers and other mobile populations in the macro system; however, the gap between migrant workers and urban workers in terms of protection level still cannot be ignored (59). Therefore, the social security system for migrant workers should be upgraded to improve their employment conditions and income, thus fundamentally improving their quality of life. Secondly, the health literacy of migrant workers should be improved by targeted health education and health promotion interventions. Finally, the development of a healthy lifestyle is crucial to improving the quality of life. Difficult working conditions, stressful work environment, and long working hours increase the probability of smoking and drinking behavior increases (60). Migrant workers are more likely to be exposed to high-intensity work and stressful conditions, rendering them vulnerable to smoking and drinking behavior. Workplace interventions should be implemented to improve the working environment of migrant workers, create a good working and living atmosphere, and guide them to develop a good lifestyle.

Some limitations of this study should be considered while interpreting the results. First, this was a cross-sectional study and the correlation between social support and quality of life was determined at a certain time-point. The study design did not permit causal inferences and the trend of changes over time could not be determined. Second, the questionnaire content information was self-reported, which may lead to reporting bias. Third, we did not consider dietary factors in lifestyle. However, unlike sleep, exercise, smoking and alcohol consumption, which vary greatly from person to person, there is no significant difference in the number and types of diets consumed by migrant workers. Finally, the study population was enrolled from urban areas in eastern China, which is economically more developed. Therefore, our findings may not be entirely generalizable to migrant workers in other less developed regions of China such as central and western China.

## 5. Conclusions

In this study, Chinese migrant workers were found to have significantly better levels of social support and quality of life than urban workers. We also observed a significant correlation between social support and quality of life, in which healthy lifestyle played a mediating role. Improving the social support and quality of life of migrant workers remains crucial. There is a need for concerted interventions by government and other related departments to strengthen social support for migrant workers. Secondly, health education and health promotion interventions for migrant workers are required to help improve their health literacy and promote healthy lifestyle leading to improved quality of life.

## Data availability statement

The original contributions presented in the study are included in the article/supplementary material, further inquiries can be directed to the corresponding author.

## Ethics statement

The studies involving human participants were reviewed and approved by Ethics Committee of Wenzhou Medical University. The patients/participants provided their written informed consent to participate in this study.

## Author contributions

FC and YY conceived and designed the research. LL, JQ, HZ, and SZ performed the data compilation and statistical analysis. YY wrote the manuscript draft. FC revised and manuscript and supervised the work. All authors contributed to the article and approved the submitted version.
